# Effects of peripheral electromagnetic stimulation after an eccentric exercise-induced delayed-onset muscle soreness protocol in professional soccer players: a randomized controlled trial

**DOI:** 10.3389/fphys.2023.1206293

**Published:** 2023-07-03

**Authors:** Hugo Keriven, Alberto Sánchez-Sierra, Diego Miñambres-Martín, Ángel González de la Flor, Guillermo García-Pérez-de-Sevilla, Diego Domínguez-Balmaseda

**Affiliations:** ^1^ Faculty of Sport Sciences, Universidad Europea de Madrid, Madrid, Spain; ^2^ Faculty of Phisioterapy and Nursing, Universidad de Castilla-La Mancha, Toledo, Spain; ^3^ Toledo Physiotherapy Research Group (GIFTO), Toledo, Spain; ^4^ Masmicrobiota Group, Faculty of Health Sciences, Universidad Europea de Madrid, Madrid, Spain

**Keywords:** soccer, football, after-treatment, muscle soreness, rehabilitation

## Abstract

**Introduction:** To examine the effects of peripheral electromagnetic stimulation in male professional soccer players on markers of Delayed Onset Muscular Soreness (DOMS), induced by a protocol of exercise (60 min of eccentric and plyometric).

**Methods:** A randomized controlled trial with fourty-five professional soccer players aged 22.33 ± 4.82 years participated in the study. Twenty-three participants were assigned to the experimental group with peripheral electromagnetic stimulation (5 stimulations of 5 s at 100 HZ with 55 s of rest for a total of 5 min of treatment) and the remaining 22 participants were assigned to the control group. Pain pressure threshold (PPT) of the vastus medialis, the Visual Analogue Scale-Fatigue (VAS-F), half squat (HS) test and the maximum voluntary contraction of the quadriceps were assessed. All evaluations were performed before and after 1 h of the eccentric exercise induced DOMS, as well as at post 24–48, and 72 h.

**Results:** Group-by-time interaction was observed in PPT of the vastus medialis (*p* = 0.040) with a medium effect size (η^2^ *p* = 0.069). From 48 to 72 h the experimental group showed an increase of PPT compared to control group (*p* = 0.015). There was no group-by-time interaction for HS, quadriceps strength and VAS-F (*p* > 0.05).

**Discussion:** Peripheral electromagnetic stimulation in male professional soccer players did not produce significant improvements in the power and strength of the lower limbs but decreased the peripheral sensitization of the vastus medialis after eccentric exercise protocol.

**Clinical Trial Registration:** https://www.anzctr.org.au/Trial/Registration/TrialReview.aspx?id=384050&isReview=true, Identifier: ACTRN12622000841774.

## 1 Introduction

Over the years, several investigations have tried to demonstrate the mechanism of appearance of the Delayed Onset Muscle Soreness (DOMS) (Sonkodi et al., 2020). DOMS can lead to a loss of joint amplitude of up to 50%, as well as a loss of muscle strength. DOMS is a common condition characterized by muscle pain, stiffness, and tenderness that typically develops 24–48 h after strenuous exercise or physical activity (Hickey et al., 2014; Hayashi et al., 2017). DOMS is caused by microscopic damage to the muscle fibers, which triggers an inflammatory response in the body. Athletes also report pain, that is, related to muscle fibers micro-damage. Some of these symptoms are related to inflammatory mechanisms. The most common is that DOMS appears when facing a return-to-play or an unusual exercise (Hickey et al., 2014; Hayashi et al., 2017). A current theory hypothesizes that the mechanism of DOMS is axonal compression. The peripheral nerves that take place in the neuromuscular spindle suffer micro-injuries, as well as a tunnel effect due to the compression generated by the muscles (Stifani, 2014; Radovanovic et al., 2015). The acute compression that could cause axonopathy may be due to the excitotoxicity of glutamate, which occurs in muscle fibers (Sonkodi, 2021). This new theory is explained in two phases: first, the damage to the neuromuscular spindle occurs, and then, there is a protective response-reflex that gives rise to symptoms such as stiffness and decreased strength (Pinho-Ribeiro et al., 2017). Clinicians try to find strategies to improve the symptoms of DOMS, to allow the return to training as soon as possible and without risks for the athletes. However, there is not a common consensus in the scientific community (Hohenauer et al., 2015; Davis et al., 2020).

In this line, peripheral electromagnetic stimulation is a non-invasive method to administer, through a rapid pulse of high-intensity electricity which causes a magnetic field in the periphery of the body, based on Faraday’s laws and experiments. Peripheral electromagnetic stimulation is a painless therapy that uses low-frequency electromagnetic waves to treat musculoskeletal conditions. Peripheral electromagnetic stimulation has been used to relieve pain, reduce inflammation, improve blood circulation, and accelerate tissue healing. This therapy has been shown to have a positive impact on various musculoskeletal conditions, including back pain, osteoarthritis, tendinitis, and postoperative pain. In this article, we will discuss the effects of peripheral electromagnetic stimulation in musculoskeletal treatment (Barker, 1991). Peripheral electromagnetic stimulation works by delivering a low-frequency electromagnetic field to the affected area of the body. This field penetrates the skin and tissues, and interacts with the cells, tissues, and nerves in the area. The electromagnetic waves cause the cells to vibrate, which in turn increases blood flow and oxygenation to the area. This increased blood flow helps to reduce inflammation and swelling and promotes tissue healing. The electromagnetic waves also stimulate the production of endorphins, which are natural painkillers produced by the body. This helps to reduce pain and improve overall comfort. One of the primary benefits of PEMS is pain relief. It has shown that PEMS can effectively reduce pain in patients with musculoskeletal conditions (Barker, 1991). The stimulation produced at the neural level is similar to conventional transcutaneous electrical nerve stimulation (TENS) (Barker, 1991), but the transcutaneous magnetic stimulation allows a deeper penetration (Kanjanapanang and Chang, 2022). In fact, peripheral magnetic stimulation triggers massive proprioceptive stimuli when applied to muscles through two pathways: direct activation of sensorimotor nerve fibers. and indirect activation of mechanoreceptors in the muscle fiber (Sdrulla et al., 2015). In stroke patients, evidence shows an increase in regional cerebral blood flow of the premotor cortex and parietal areas in the injured hemisphere after applying peripheral magnetic stimulation to the paretic muscles (Meesen et al., 2011; Beaulieu and Schneider, 2015).

Peripheral electromagnetic stimulation tends to improve recovery through the new sensory input that it manages to generate. Said stimulation acts on the peripheral nerve, in our case the femoral nerve, being able to counteract the nociceptive signal that comes out of the axonal endings found in the muscle. Peripheral electromagnetic stimulation has an effect on the A beta fibers that are responsible for acute pain signals. It also achieves an improvement in the response, through a maximum recruitment of fibers with minimal skin stimulation, that is, involved in the process of an increase in strength and with it, in the improvement of recovery (Asao et al., 2022; Jiang et al., 2022).

Based on these findings and in order to improve the symptoms of muscle soreness, this study aimed to observe the effects of peripheral electromagnetic stimulation on performance and recovery in young professional soccer players suffering after an eccentric exercise-induced delayed-onset muscle soreness protocol. We hypothesized that the peripheral electromagnetic stimulation have positive effects in pressure pain threshold values of the vastus medialis, perception of fatigue during half squat, half squat speed and quadriceps peak force.

## 2 Methods

### 2.1 Study design

A double-blind randomized pilot study was carried out with professional soccer players, following the ethical principles of the Declaration of Helsinki. This study was elaborated following the Consolidated Standards of Reporting Trials (CONSORT) and was approved by the Research Ethics Committee of the (reference number: CIPI: 22.095) and registered (ACTRN12622000841774). The double blind allowed neither the examiner nor the data analyst to know the allocation to each of the situations.

### 2.2 Sample size calculation

For the sample size calculation, an alpha error of 0.05 and a beta error of 0.2 and medium effect size (f = 0.25 or Eta partial squared = 0.06) were determined. Therefore, using the G*Power (3.1.9.2) software, at least of 34 participants were required to achieve our objective. To account for an estimated dropout rate of 10%, the required sample size was adjusted to 38 participants, divided into two groups (n = 19).

### 2.3 Participants

In order to be part of the study, the participants had to meet the inclusion criteria: 1) male subjects aged 18 and 35 years old; 2) not have suffered a lower limb muscle injury in the past 6 months; 3) not have undergone surgery in the past 12 months of the lower limbs; 4) not presenting any contraindication regarding the application of electrotherapy. Once participants were enrolled in the study, demographic data (age) and anthropometric (weight and height) data were recorded. The weight (kg) was measured with a scale (Ano Sayol SL, Barcelona, Spain) and the height (cm) with a stadiometer (Asimed T2, Barcelona, Spain). The body mass index (BMI) was calculated as weight (kg)/height2 (m). Duration and number of training sessions was recorded.

The participants were randomized to the intervention group or the placebo group using the random function of Microsoft Office Excel (Microsoft Corporation, Redmond, Washington, United States). Prior to be included in the study, all participants signed informed consent.

### 2.4 Procedure

**FIGURE 1:**
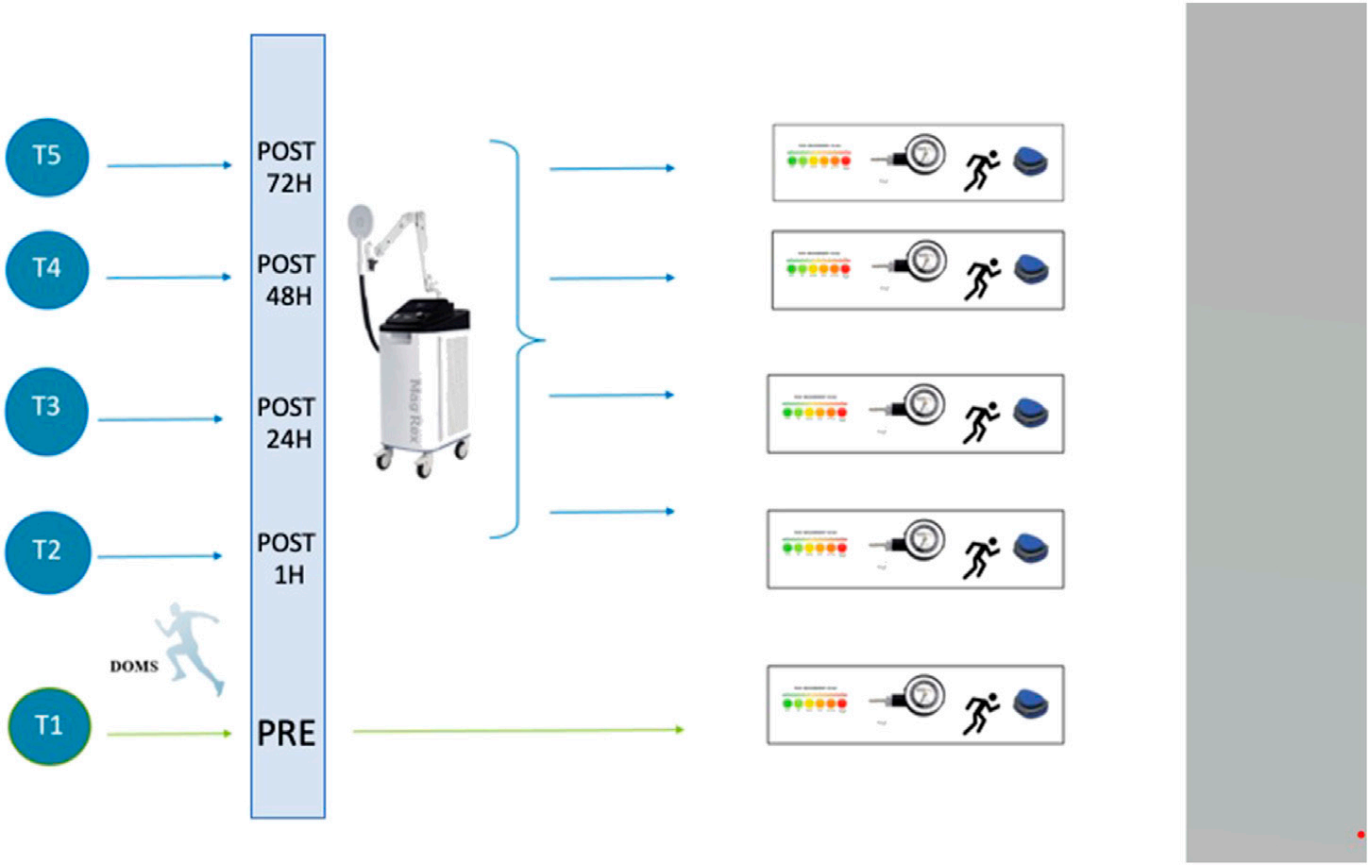
Study design and intervention schedule. Pre-Post: times before, after 1 h, 24 h, 48 h, and 72 h of eccentric exercise-induced delayed-onset muscle soreness.

The participants were submitted to five evaluation sessions throughout the study, as illustrated in Figure 1.

All participants have a familiarization session 1 week before the first session of the study. On the first session (day one, pre muscle damage protocol) anthropometric, creatine kinase, blood lactate levels, half squat speed, maximal voluntary contraction of the quadirceps, fatigue perception and PPT measurements were performed. On the first session (1 h after), 24, 48 and 72 h after the muscle damage protocol the same measurements were recorded. In order to examine the effects of the muscle damge protocol the creatine kinase and lactate levels were recorded. Creatin kinase (CK) measurement was performed using venous blood obtained by venipuncture. A diagnostic instrument designed to analyze blood parameters using test strips called Reflotron Plus (Roche Diagnostics, Barcelona, Spain) was used following the study by Baird et al. (2012). Participants were reminded not to engage in physical activity for at least 2 days prior to the study, as this enzyme may rise days after physical exercise. Lactate levels were obtained from venous blood following the protocol of Clemente et al. (2010). Blood lactate concentrations were measured by electroenzymatic analysis (Lactate Scout Pro, Musimedic S.L. Donostia, Spain).

### 2.5 Intervention

#### 2.5.1 Eccentric exercise protocol

All the participants performed the eccentric exercises session for 60 min. The protocol was based on the study performed by Howatson et al. (2012). They previously performed a warm-up with the physical trainer based on ballistic, mobility, strength, and running exercises. The 60-min session included performing 10 sets of 10 repetitions of vertical countermovement high jump from a 40-cm-high step (drop jump), carrying 10% bodyweight dumbbells, with a 1-min rest between sets, and 15 s between each repetition. In addition, a circuit of eccentric exercises was added, which is detailed below. After the plyometric exercise, 3 sets of the 3 exercises described below were performed: 1) Lower limbs rear kick (one-leg deadlift) hanging 10 kg of weight on the chest, performing 3 sets of 10 repetitions with each leg. 2) Suspension race simulation, performing 3 sets of 20 s 3) Nordic hamstring curl, performing 3 sets of 10 repetitions.

#### 2.5.2 Peripheral electromagnetic stimulation

After the induced DOMS session, the participants received a peripheral electromagnetic stimulation treatment with a Long-Term Potentiation (LTP) program described below, in the case of being part of the experimental group (EG). As for the control group (CG), the machine was placed 20 cm far from the skin to make sure that the effect could not reach it, and with a lower intensity so that the participants heard the same noise as that of their teammates in the EG, but without finding an effect.

This treatment was based on the studies of Sdrulla et al. about the improvement of synapses with the use of pulse trains of 100 HZ on the Aβ fibers. This stimulation also produces an effect at the central level (Beltrá et al., 2022). It consisted of 5 stimulations of 5 s at 100 HZ with 55 s of rest for a total of 5 min of treatment (Sdrulla et al., 2015). It was applied every day during the 3 days just before the measurements. The probe was placed on the inner side of the thigh near the femoral nerve to act on the entire quadriceps.

### 2.6 Outcome measures

#### 2.6.1 Primary outcome measure

##### 2.6.1.1 Pressure pain threshold

The protocol and methodology of the study was conducted following previously performed by Fleckenstein et al. (2017). It is currently known that the pressure pain threshold (PPT) is a valid and reliable method to assess mechanical threshold. It can be defined as the minimum pressure necessary to produce pain. Before starting the eccentric exercises, measurements had to be made to verify that the participants did not experience pain on palpation and the concept of PPT was explained to them, as “the moment in which the pressure stimulus changes to a sensation of pressure at pain”. The applied pressure was 1kg/cm2, until the subjects reported the onset of pain. In this study a Baseline algometer (Wagner instruments, Greenwich, United States) was used.

#### 2.6.2 Secondary outcome measures

##### 2.6.2.1 Visual Analogue Scale—Fatigue

The Visual Analog Scale to evaluate fatigue (VAS-F) was used to evaluate fatigue during the HS test (Nadarajah et al., 2017). Before carrying out the performance analysis test, the participants were informed that they would be asked at the end of the jump test the amount of pain that they could feel during the execution of the half squat (HS) test. When doing so, it is necessary to show them a 100 mm horizontal line, of this size to better appreciate the changes throughout the study, that the extremes represent the extremities of the evaluated fatigue: “0”will be the absence of fatigue and “100” an unbearable fatigue. The participants had to indicate their perception of fatigue after performing the HS test. This instrument has showed an excellent internal consistency (Cronbach alpha = 0.90) (Nadarajah et al., 2017).

##### 2.6.2.2 Half Squat

Lower body performance was analyzed with a HS test consisting of maximum power effort. A warm-up was previously carried out for the familiarization of the exercise, for the angulation of the hip and knee joint. The HS was previously performed at 50% of the participant’s body weight, then the missing kilograms were added to reach 80% of the body weight of each subject. Next, the maximum mechanical power developed with an added load (80% of body weight) was evaluated with the HS exercise. They had to perform 2 repetitions with 3 min of rest between them. To fix the load that corresponds to 80% of each subject, it was necessary to carry out a test on the first day in the gym. This load was then maintained throughout the study. When perfoming the HS test, participants stand in a standing position with their feet hip-width and shoulder-width apart. The hands must be gripped to the bar, which was supported later by the cervical spine, at the C5-C7 level on the trapezius and rear deltoids, with a protective ergonomic cushion. Participants must flex their knees to 90° and then proceed to full power extension to return to the original standing position (Bogdanis et al., 2014). The test was carried out at maximum speed on a Smith Multipower bar guide machine, using 20, 10, 5, 2.5 and 1.25 kg discs (Technogym, Gambettola, Italy). By having both ends fixed, this allows only the movement of the bar, vertical in this case. To have an estimation of the execution speed of each subject in each repetition, the test was carried out with an Encoder of the ADR encoder brand (Toledo, Spain). It is held attached to one end of the bar so as not to disturb the movement of the HS. Thus, it allows to measure the vertical displacement of the bar in terms of the movement of the exercise, implementing the software of the ADR system (Toledo, Spain), and recording the speed of the bar in m/s (average and peak).

##### 2.6.2.3 Quadriceps strength

A hand-held dynamometer (Activbody, San Diego, CA) (Thorborg et al., 2009) was used to assess maximum voluntary isometric contractions of quadriceps (Hung et al., 2021). A total of three repetitions with the maximum power of each participant was measured (Hung et al., 2021). The participants were seated on the edge of a physiotherapy table with the knee at 90 degrees of flexion, and the dynamometer was placed at the level of distal tibia (Hung et al., 2021). Then, the maximum peak force during each contraction was sought with a normalization against the weight of each participant (Rezaei et al., 2014). There was a rest of 3 minutes between measures (Babault et al., 2003; Rezaei et al., 2014). At the end of the measurement, the participants rested for 3 min before continuing with the other measurements.

### 2.7 Statistical analysis

Statistical analyses were carried out using the SPSS v.25 program (IBM, Armonk, NY, United States). The normal distribution of the data was verified using the Shapiro-Wilk test and histograms. *p* values < 0.05 were considered as non-normally distributed and *p* > 0.05 as normally distributed a descriptive analysis was conducted to characterize the sample. Central tendency and dispersion data were reported as mean and standard deviation for normal-distributed variables, or as median and interquartile range for non-normal-distributed variables, respectively. For the quantitative variables, independent t-test or Mann-Whitney U test were carried out to compare the means groups at baseline. Homoskedasticity and sphericity were checked. When the assumptions were met, a mixed analysis of variance (ANOVA) 5 × 2 was carried out, adjusting multiple comparisons with the Bonferroni test. The effect size was estimated with partial eta squared (η 2), interpreting values of .01, .06, .14 as small, medium and large, respectively. A 95% confidence interval was followed (Adams et al., 2021).

## 3 Results

### 3.1 Baseline data

**FIGURE 2:**
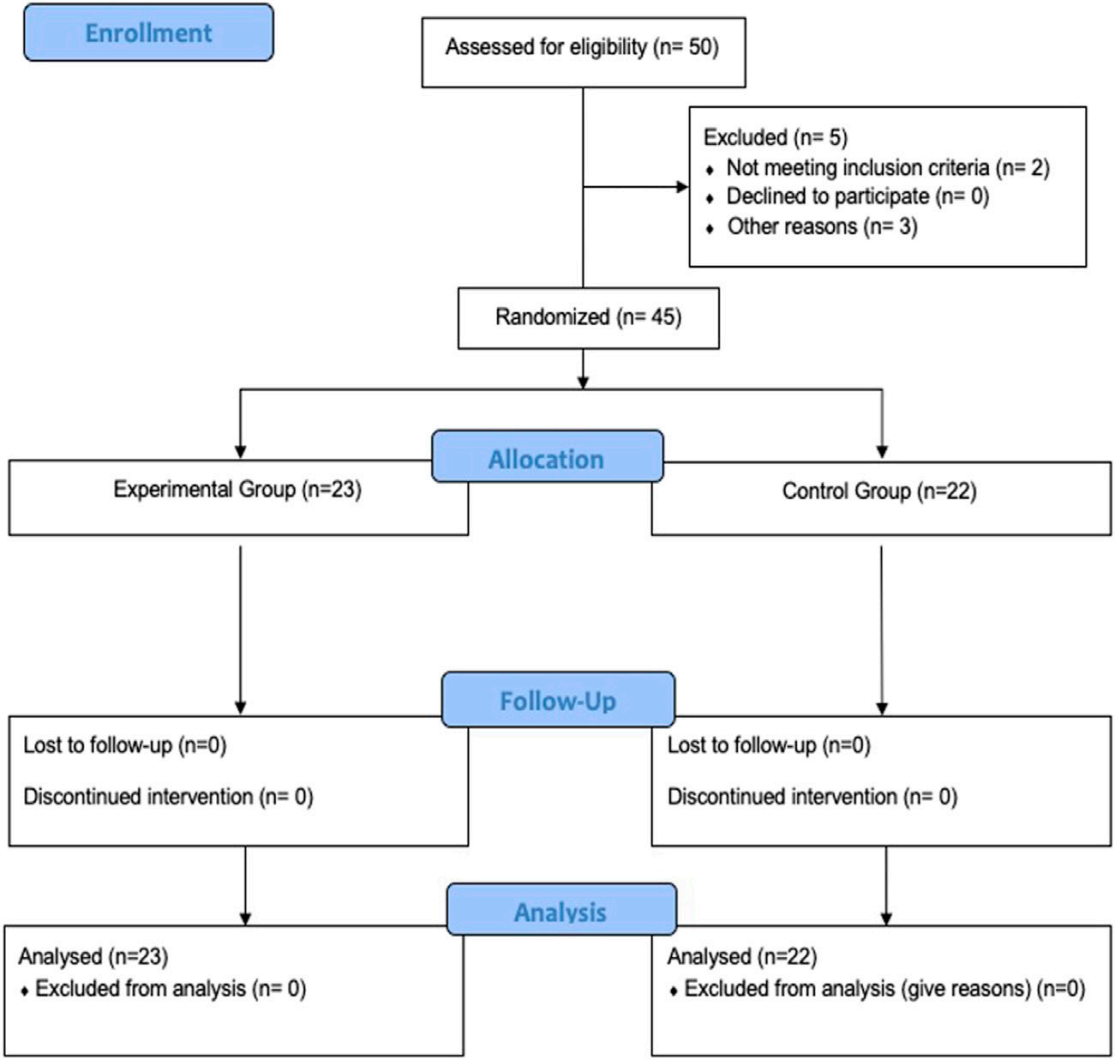
Study flowchart.

Out of a total of 50 volunteers willing to participate in the study, 5 were excluded (Figure 2). Therefore, a total of 45 male participants aged 22.33 ± 4.82 years were included and analyzed in the study. The anthropometric characteristic did not differ between participants of both groups (Table 1). All the participants trained five times a week for 90 min.

**TABLE 1:** Sociodemographic characteristics and pre-treatment scores of the total sample, experimental and control group.

Variables	Total sample (n = 45)	EG (n = 23)	CG (n = 22)	*p*-value (between-group)
Age (years)	22.33 ± 4.42	22.87 ± 4.94	21.77 ± 3.84	0.412
Height (cm)	178.67 ± 6.88	178.39 ± 7.27	178.96 ± 6.62	0.787
Weight (kg)	74.14 ± 8.83	74.76 ± 8.29	73.50 ± 9.52	0.636
Body mass index (kg./m2)	23.21 ± 2.24	23.48 ± 2.13	22.92 ± 2.36	0.405
Soccer practice experience (years)	14.52 ± 5.6	14.54 ± 5.56	16.12 ± 3.83	0.890

CG, control group; EG, experimental group. Results are expressed as mean ± standard deviation

**FIGURE 3:**
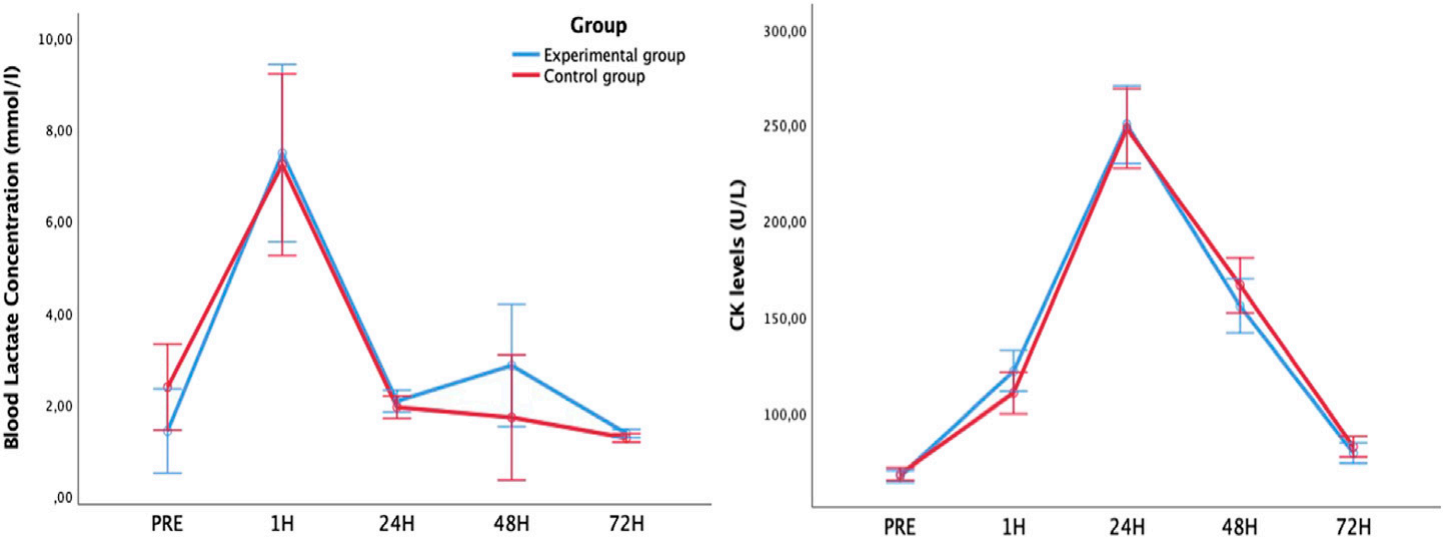
Blood Lactate (left) and creatin kinase (right) concentration levels evolution after eccentric exercise-induced delayed-onset muscle soreness comparing experimental and control group.

To control that the response to the eccentric exercise-induced DOMS session was homogeneous for both groups, the CK and blood levels of lactate were measured. An increased of CK was observed 1, 24, 48 and 72 h after intervention in both groups (*p* < 0.001) and no interaction between groups was detected in pre (*p* = 0.589), post 1 h (*p* = 137), post 24 h (*p* = 0.891), post 48 h (*p* = 0.284) and post 72 h (*p* = 0.379). An increased of blood lactate levels was observed 1 h after intervention in both groups (*p* < 0.001) and no interaction between groups and time was observed (*p* = 0.814) (Figure 3).

**FIGURE 4:**
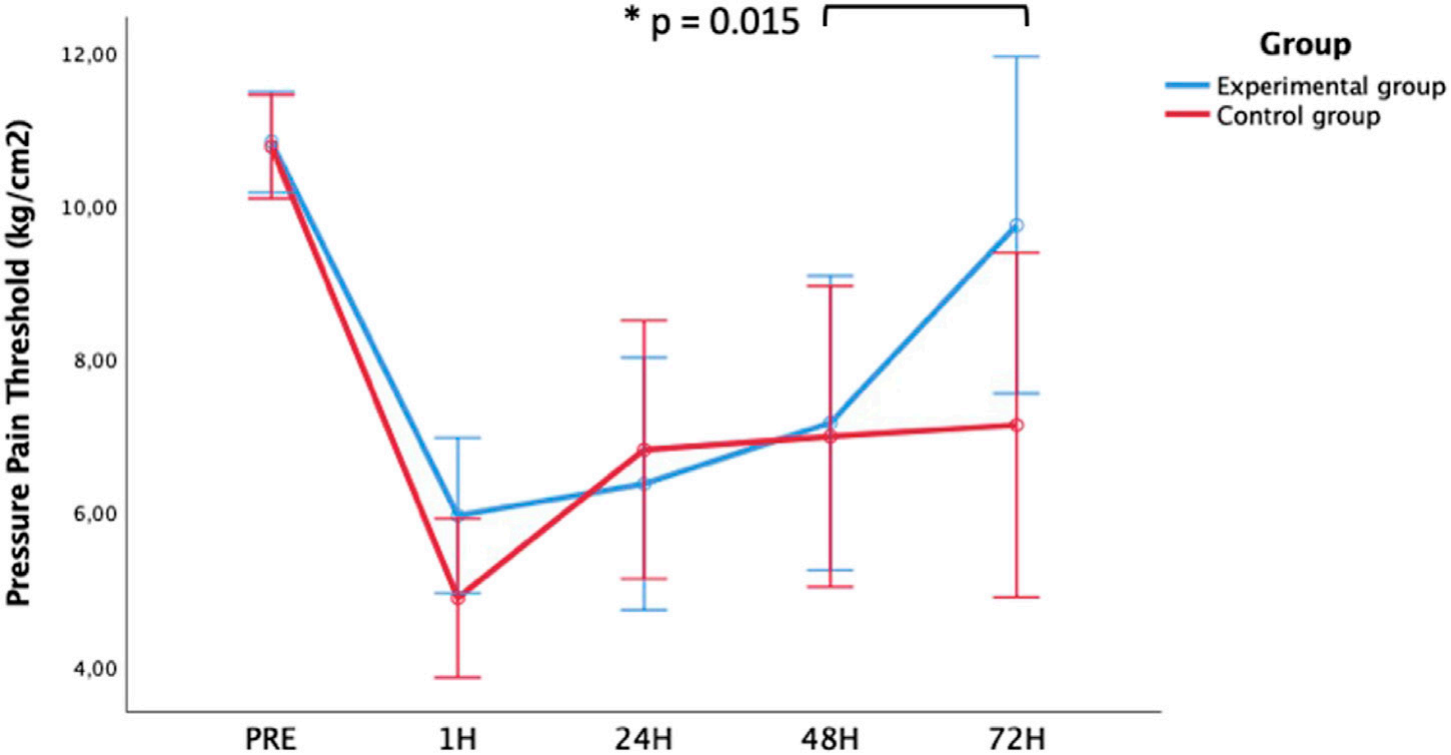
Blood Lactate (left) and creatin kinase (right) concentration levels evolution after eccentric exercise-induced delayed-onset muscle soreness comparing experimental and control group.

Baseline measures of the dependent variables did not show significant differences between experimental and control group (Table 2). There was no group-by-time interaction for HS, (*p* = 0.380), quadriceps strength (*p* = 0.652) and VAS-F (*p* = 0.864). Group-by-time interaction was observed in PPT of the vastus medialis (*p* = 0.040) with a medium effect size (η^2^_p_ = 0.069) (Table 2). From 48 to 72 h the experimental group showed an increase of PPT compared to control group (*p* = 0.015) (Figure 4). Time effect was observed for HS, quadriceps strength, VAS-F and PPT (*p* < 0.001). Despite a progressive recovery of maximal strength of the quadriceps after the muscle damage protocol, HS speed, PPT of the vastus medialis did not return to baseline values within 72 h (Table 2; Figure 4).

**TABLE 2:** Differences between the experimental and control groups for the dependent variables.

	T1 (baseline)	T2 (post 1 h)	T3 (post 24 h)	T4 (post 48 h)	T5 (post 72 h)	p (time x group)	η^2^_p_
Primary Outcome Measure							
PPT (kg/cm2)							
CG (n = 22)	10.77 ± 1.47	4.89 ± 2.61	6.82 ± 4.62	5.99 ± 5.22	7.14 ± 4.58	0.040	0.069
EG (n = 23)	10.83 ± 1.65	5.96 ± 2.19	6.37 ± 3.09	7.17 ± 3.83	9.74 ± 5.76		
Secondary Ourcome Measures							
Quadriceps Strength (Kg)							
CG (n = 22)	43.99 ± 14.23	36.84 ± 12.45	42.75 ± 12.15	47.22 ± 11.21	49.60 ± 11.76	0.652	0.010
EG (n = 23)	47.96 ± 11.73	39.04 ± 10.91	44.59 ± 11.19	47.96 ± 12.58	50.98 ± 13.07		
VAS-F (mm)							
CG (n = 22)	0.00 ± 0.00	49.59 ± 16.95	44.32 ± 20.84	34.68 ± 22.87	21.23 ± 15.00	0.864	0.007
EG (n = 23)	0.00 ± 0.00	50.91 ± 16.67	43.48 ± 14.65	37.30 ± 19.83	26.09 ± 18.09		
Half Squat (m/s)							
CG (n = 22)	0.81 ± 0.11	0.68 ± 0.12	0.73 ± 0.11	0.77 ± 0.12	0.80 ± 0.11	0.365	0.024
EG (n = 23)	0.85 ± 0.12	0.74 ± 0.08	0.75 ± 0.10	0.81 ± 0.14	0.86 ± 0.11		

CG, control group; EG, experimental group; PPT, pain pressure threshold; VAS, visual analogic scale to evaluate fatigue; η^2^_p_, partial eta squared.

## 4 Discussion

This novel study aimed to observe the influence of peripheral electromagnetic stimulation on performance and recovery in professional soccer players suffering from induced-DOMS. Peripheral electromagnetic stimulation aimed to improve the PPT in the quadriceps, compared to control group. There were no significant differences between groups and time in the rest of the variables analyzed.

Including eccentric exercise sessions that cause DOMS in training dynamics is necessary in sports to improve performance (Cheung et al., 2003). Several articles have been published that explore the strategies offered by electrotherapy by applying a TENS for recovery that aims to accompany the athlete and minimize indirect markers of muscle damage (Craig et al., 1996; Butera et al., 2018). Our study evaluated the effects of applying the LTP protocol in males who suffered from DOMS induced by a 1-h session of eccentric strength exercises, never tested to date.

According to the results, the group treated with the peripheral electromagnetic stimulation current did not improve the muscle recovery in terms of performance in the 72 h after performing a series of exercises with large eccentric component that generated DOMS, compared to the control group. These findings would indicate that the muscular responses that follow this type of acute exercises that cause DOMS were not altered by the treatment, since in both groups showed a decrease in performance in the measurements made in the post exercise/T2. The improvements observed in the PPT of the vastus medialis could have been influenced by other mechanisms, such as peripheral sensitization (Amiri et al., 2021; García-Pérez-de-Sevilla et al., 2022).

First, pressure pain threshold was analyzed with an algometer at the vastus medialis of the quadriceps, revealing a significant difference between groups favoring the EG, with a moderate effect size, even considering that there is a physiological process where after 48 h the PPT is usually higher due to the regeneration processes that are taking place in the damaged fibers (Fleckenstein et al., 2017). In addition, there was a significant difference between groups in the PPT of the vastus medialis comparing T4 and T5 favoring the peripheral electromagnetic stimulation (*p* = 0.015), as has been observed in other studies (Sdrulla et al., 2015). Peripheral electromagnetic stimulation also has anti-inflammatory effects. Inflammation is a natural response of the body to injury or infection, but when it becomes chronic, it can cause tissue damage and contribute to the development of musculoskeletal conditions. Peripheral electromagnetic stimulation has been shown to reduce inflammation in several ways. First, it increases blood flow to the affected area, which helps to remove inflammatory chemicals from the tissue. Second, it stimulates the production of anti-inflammatory cytokines, which help to reduce inflammation. Finally, peripheral electromagnetic stimulation has been shown to decrease the production of pro-inflammatory cytokines, which contribute to the development of chronic inflammation (Barker, 1991).

In the analysis of the perception of fatigue measured with the VAS-F scale, no significant differences were found between groups. Fan and Sdrulla showed that distal hypoalgesia is not seen in all the stimuli that athletes may face when training (Fan and Sdrulla, 2020). In addition, other muscle groups apart from the quadriceps could have had an influence on the pain perception described by the participants.

Regarding the HS test, there were no significant differences between groups, which may be due to the synergy of other muscles that are involved in performing this test. Similar findings were reported in the study performed by Goulart et al. in which they suggested that the interval between sessions could influence the performance of explosive actions, although the HS test is a validated tool to estimate the power of the lower limbs (Goulart KN de et al., 2021). On the other hand, in the study conducted by Dominguez-Balmaseda et al. the EG maintained the power of the lower limbs in HS after performing a protocol of eccentric exercises that induce DOMS, especially in the post 24 h. It should be noted that the study carried out by Dominguez-Balmaseda et al. had a larger sample size, and the treatment consisted of herbal supplementation (Dominguez-Balmaseda et al., 2020). Although in the present study there were no significant between-group differences in the power of the lower limbs, there were significant improvements in the EG in the within-group analysis, suggesting that it would be interesting to perform future studies with a larger sample size to see if this therapeutic strategy could improve DOMS symptoms and with it, sports performance.

Concerning the quadriceps strength, there were not significant differences between groups, but this could be influenced by the loss of strength that the test entails if the participant approaches the extension (Babault et al., 2003). However, there were significant differences in the within-group analysis for the EG, suggesting that the peripheral electromagnetic stimulation current should be further investigated. Other post-exercise recovery strategies such as electrostimulation or protein ingestion has been proposed. Recently, Hartono et al. (2022) have performed a systematic review including 84 trained young males and ten longer-term studies including 167 trained and 391 untrained participants. They conclude that protein ingestion enhanced myofibrillar protein synthesis rates, but not mitochondrial protein synthesis rates during post-exercise recovery after an acute bout of concurrent exercise demonstrated in acute studies. Of the included longer-term training studies, five out of nine reported that protein supplementation enhanced concurrent training-mediated increases in muscle mass, while five out of nine studies reported that protein supplementation enhanced concurrent training-mediated increases in muscle strength and/or power. Therefore, the peripheral electromagnetic stimulation may not increase the muscle strength or peak force after an eccentric exercise-induced delayed-onset muscle soreness protocol compared to the control group. Protein ingestion or other potential recovery strategies could be enhanced to improve the performance in soccer players.

### 4.1 Limitations

The present study has several limitations. A key factor that we should consider is that the usual load of the training sessions followed by the participants could influence their recoveries as they are used to higher intensity work compared to the exercises performed in our study. Finally, the findings of the present study cannot be extrapolated to female or sedentary population. Therefore, it would be interesting to evaluate the efficacy of our treatment in other types of populations like female participants or less active populations.

In future studies, parameters such as heart rate variability or the Counter Movement Jump test should be evaluated, due to the already demonstrated implication that both have on recovery and anticipation by athletes. In addition, parameters such as electromyography (EMG) could be added in future research to be able to analyze muscle recruitment after peripheral stimulation, to determine the real impact of peripheral stimulation on the peripheral nervous system (Beltrá et al., 2022). It would be interesting in future studies to interact with athletes of the same competitive level at the beginning of the season in order to see if the daily training load influences recovery. Clemente et al. carried out a review in 2021 where they observed that soccer players present lower physical performance at the beginning of a pre-season. Thus, it would be interesting if future studies are developed in the early stages of a return to training (Clemente et al., 2021). In addition, it would be interesting to add the 30-m run, since it is a common sprint distance in soccer, to deepen the analysis of the influence of peripheral stimulation on physical performance (Pearcey et al., 2015).

### 4.2 Practical application

Peripheral electromagnetic stimulation is a safe and effective therapy for musculoskeletal conditions. It can relieve pain, reduce inflammation, improve circulation, and promote tissue healing. Peripheral electromagnetic stimulation can be used alone or in combination with other therapies, such as physical therapy, cryotherapy or supplementation.

## 5 Conclusion

Peripheral electromagnetic stimulation in male professional soccer players did not produce significant improvements in the power and strength of the lower limbs but decreased the peripheral sensitization measured by pressure pain threshold of the vastus medialis after an eccentric exercise-induced delayed-onset muscle soreness protocol.

## Data Availability

The raw data supporting the conclusion of this article will be made available by the authors, without undue reservation.
